# The interplay between surfaces and soluble factors define the immunologic and angiogenic properties of myeloid dendritic cells

**DOI:** 10.1186/1471-2172-12-35

**Published:** 2011-06-06

**Authors:** Leslee Sprague, Maria Muccioli, Michelle Pate, Evan Meles, John McGinty, Harika Nandigam, Amritha K Venkatesh, Ming-yu Gu, Kristen Mansfield, Andrew Rutowski, Omowaleola Omosebi, Maria C Courreges, Fabian Benencia

**Affiliations:** 1 Biomedical Engineering Program, Russ College of Engineering and Technology, Ohio University, USA; 2 Molecular and Cell Biology Program, Ohio University, USA; 3 Department of Biomedical Sciences, College of Osteopathic Medicine, Ohio University, USA

## Abstract

**Background:**

Dendritic cells (DCs) are antigen presenting cells capable of inducing specific immune responses against microbial infections, transplant antigens, or tumors. Interestingly, microenvironment conditions such as those present in tumor settings might induce a DC phenotype that is poorly immunogenic and with the capability of promoting angiogenesis. We hypothesize that this plasticity may be caused not only by the action of specific cytokines or growth factors but also by the properties of the surfaces with which they interact, such as extracellular matrix (ECM) components.

**Results:**

Herewith we studied the effect of different surfaces and soluble factors on the biology of DCs. To accomplish this, we cultured murine myeloid(m) DCs on surfaces coated with fibronectin, collagen I, gelatin, and Matrigel using poly-D-lysine and polystyrene as non-biological surfaces. Further, we cultured these cells in the presence of regular DC medium (RPMI 10% FBS) or commercially available endothelial medium (EGM-2). We determined that mDCs could be kept in culture up to 3 weeks in these conditions, but only in the presence of GM-CSF. We were able to determine that long-term DC cultures produce an array of angiogenic factors, and that some of these cultures still retain the capability to induce T cell responses.

**Conclusions:**

Altogether these data indicate that in order to design DC-based vaccines or treatments focused on changing the phenotype of DCs associated with diseases such as cancer or atherosclerosis, it becomes necessary to fully investigate the microenvironment in which these cells are present or will be delivered.

## Background

Dendritic cells (DCs) are professional antigen presenting cells (APCs) found in peripheral tissues and in immunological organs such as thymus, bone marrow, spleen, lymph nodes and Peyer's patches [1-3]. In the mouse, DCs can be broadly divided into plasmacytoid and myeloid DCs [4]. Plasmacytoid DCs (pDCs) are characterized by the expression of B220 but no CD11b and produce large amounts of type-1 interferon in response to viral infections [5,6]. On the other hand, bone marrow-derived DCs (myeloid DCs) are present in most tissues and are characterized by coexpression of CD11c and CD11b markers. As reviewed by Breckpot *et al. *(2009), these DCs respond to GM-CSF and are capable of producing IL-12 in response to toll-like receptor ligands. Interestingly, DCs have been shown to possess a remarkable cellular plasticity. For example, pDCs could acquire myeloid DC characteristics under the influence of viral infection [5].

In order to elicit productive T cells responses, DC major histocompatibility (MHC)/peptide complexes must interact with specific T cell receptors (Signal 1) in the context of an appropriate costimulatory molecule interaction between both cell types (Signal 2). It has been recently considered that the microenvironment where this interaction occurs (Signal 3) will determine the fate the subsequent immune response towards an immunogenic or tolerogenic response [4]. A clear example of the relevance of the microenvironment on DC biology can be observed in tumor settings. Molecules present in the tumor milieu such as vascular endothelial growth factor (VEGF), interleukin (IL)-10 and prostaglandin-2 (PGE-2) can profoundly affect the biology of DCs making them immunosuppressive, incapable of inducing specific immune responses or capable of inducing regulatory T cells [7,8]. In particular, DCs showing low levels of costimulatory molecules have been detected in microenvironments characterized by high levels of VEGF [9]. These DCs, showing highly immunosuppressive properties, are able to render T cells anergic or tolerised, thus abrogating immune responses. On the contrary, endothelial cell-produced antiangiogenic cytokine vascular endothelial growth inhibitor induces DC maturation [10]. Furthermore, treatment of the tolerogenic DCs with inflammatory molecules, render immunogenic DCs with the capability to activate T cells [11]. Besides an immune "paralysis", we and others have shown that DCs, or leukocytes expressing DC markers are able to produce angiogenic factors and can promote angiogenesis [12-15].

We hypothesized that this plasticity might be caused not only by the action of specific cytokines or growth factors, but also by the interaction of these cells with extracellular matrix (ECM) components. Herewith, we performed a series of studies in order to determine the influence of different surfaces and growth factors on the biological properties of myeloid DCs.

## Methods

### Animals

Six to eight week old female C57BL/6 (H-2Kb) and BALB/c (H-2Kd) mice (Charles River Laboratories, Wilmington, MA) were used in protocols approved by the Institutional Animal Care and Use Committee at Ohio University.

### *In vitro *generation and maturation of murine DCs

Murine DCs were generated from bone marrow precursors recovered from femurs and tibiae of 6-8 week old female C57BL/6 mice by the method of Lutz *et al. *[16,17]. Briefly, bone marrow cells were dispersed by vigorous pipetting and cultured in RPMI-1640 supplemented with penicillin (100 μg/ml), streptomycin (100 U/ml), L-glutamine (2 mM) and 10% heat-inactivated fetal bovine serum (FBS) (all Invitrogen, Carlsbad, CA) in the presence of 20 ng/ml of recombinant mouse granulocyte-macrophage colony-stimulating factor (GM-CSF, 315-03, Peprotech Inc., Rocky Hill, NJ) for 8 days. GM-CSF was replenished on days 3 and 6. In some experiments, maturation was induced by culturing the cells for 2 days in the presence of 5 ng/ml GM-CSF, 20 ng/ml mouse tumor necrosis factor alpha (TNF-α, 315-01A, Peprotech) and 100 ng/ml bacterial lipopolysaccharide (LPS from E. coli, serotype 0111:B4, L2630, Sigma).

### Cell lines and tumors

In some experiments we used the murine ID8-Vegf-A cell line of ovarian cancer [18]. The ID8 cell line is a tumor cell line derived from spontaneous *in vitro *malignant transformation of C57BL/6 mouse ovarian surface epithelial cells originally generated by Roby *et al. *[19]. This line has been engineered to express high levels of VEGF-A (VEGF-164) [20]. These cells were maintained in DMEM supplemented with 2 mM L-glutamine, 100 μg/ml penicillin, 100 U/ml streptomycin, and 10% heat-inactivated fetal bovine serum (FBS) (all Invitrogen). Ectopic ID8-Vegf-A solid ovarian tumors were initiated in C57BL/6 mice by subcutaneous injection of 7 × 10^6 ^tumor cells [20,21].

### Culture of DCs on different surfaces

In order to investigate the effect of different surfaces on the biology of DCs, these cells were seeded on commercially available 6-well plates coated with different extracellular matrix components such as fibronectin, collagen I, gelatin, Matrigel or synthetic poly-D-Lysine (all BD Biosciences, San Jose, CA). Controls included cells cultured on regular polystyrene tissue culture plates (Corning Costar, Corning, NY). DCs were seeded on these plates at a concentration of 5 × 10^5 ^cells/ml in either RPMI 10% FBS or endothelial EBM-2 medium with supplements (EGM2-MV BulletKit, Lonza) with or without the addition of GM-CSF (3 ng/ml). Cells were cultured up to 3 weeks in these conditions and media was replenished once a week. Pictures of live cells were obtained with an inverted microscope attached to a Motic 2000 Camera (Motic, Richmond, British Columbia, Canada). In order to perform specific studies, after different times in culture cell supernatants were collected, surfaces washed with PBS and attached cells recovered by using cell scrapers.

In order to investigate the effect of tumor-derived GM-CSF on DCs, we prepared tumor conditioned medium. To accomplish this, ID8-Vegf-A cells were cultured until 80% confluence and supernatants collected and filtered. Then, these supernatants were mixed with RPMI medium (30:70; conditioned medium:RPMI) and used to culture DCs for 1 week. As a control of GM-CSF specificity we also cultured these cells in the presence of anti-GM-CSF receptor antibody (rabbit polyclonal, sc25472, Santa Cruz Biotechnology Inc. Santa Cruz, CA) as previously described [22] or isotype control.

### Purification of CD11c by means of magnetic sorting

In some experiments CD11c cells were recovered from the cultures by magnetic sorting. To accomplish this, cells were mechanically detached from the culture plates by using cell scrapers. After blocking Fc receptors with anti-CD16/CD32 antibody (Fc block, 2.4G2; BD Pharmingen, San Diego, CA), cells were labeled with anti-CD11c magnetic beads (MACS Miltenyi, Auburn, CA) and positive cells isolated by using MS paramagnetic columns in an octoMACS magnet (all MACS Miltenyi) following the manufacturer's instructions.

### Flow cytometry

Cells were subjected to three-color flow cytometry on a FACSort flow cytometer using CellQuest 3.2.1f1 software. (Becton Dickinson, San Jose, CA). We collected 10.000 events per sample. Non-specific staining was blocked with Fc block in FACS buffer (PBS with 2% FBS and 0.05% sodium azide). Fluorochrome-conjugated monoclonal antibodies against CD45 (30-F11), CD11c (HL3), CD80 (16-10A1), CD86 (GL1), MHC-II (KH74), CD54 (3E2), CD11b (M1/70), GR1 (RB6-8C5), CD40 (3/23), PSGL-1 (2PHI), (all BD Biosciences, San Diego, CA); CD29 (HMb1-1), CD49b (DX5), CD49d (R1-2), CD49e (HMa5-1), CD49f (GoH3), CD41 (MWReg30), CD51 (RMV-7), CD61 (2C9.G3), OX40-L (RM134L), CD44 (IM7), CD18 (M18/2), (all eBioscience, San Diego, CA); and CD49a (HMalpha1, AbD Serotec, Raleigh, NC) were used at 1/100 dilution.

### RT-PCR and Real-Time Quantitative Reverse Transcription-PCR

RNA was isolated with TRIzol (Invitrogen) and then reverse transcribed by using the High Capacity cDNA Reverse Transcription Kit (Applied Biosystems, Foster City, CA) following the manufacturer's instructions. All RNA samples were treated with DNAse in order to eliminate possible contaminating genomic DNA. For qualitative PCR analysis, the PCR cycling was conducted with Taq polymerase at 94°C (30 s), 57°C (30 s), and 72°C (20 s) for 40 cycles. Expression of specific molecules was also analyzed at the level of RNA by means of quantitative real time RT-PCR (qPCR) analysis. For qPCR experiments, we used the absolute quantification method by generating standard curves for our genes of interest, and housekeeping gene. We normalized the cDNA load to mouse glyceraldehyde-3-phosphate dehydrogenase (GAPDH). Data were expressed as relative units to GAPDH mRNA molecules. In these assays, we used (PerfeCTa SYBR Green FastMix, Quantas Biosciences) for detection of the PCR reaction. Each amplification experiment was performed in 96-well optical grade PCR plates covered with optical film) in an iCycler iQ5 real-time PCR instrument (Bio-Rad Laboratories, Hercules, CA).

Primers are described in Table 1. All the primers were designed with the public web Primer 3 program in order to generate PCR products that cross introns and ranging between 85 and 115 bp of length. We have used this program to design the primers described in our previous publications [12,21,23,24].

**Table 1:** List of primers used for qualitative and quantitative PCR studies.

Target Gene	Primer Sequence
CD29	Forward 5'-CAA ATG CCA AAT CTT GCG GAG A-3'
	Reverse 5'-GCA TTC CTT CTT GCA AAA ATG TCG T-3'

CD49a	Forward 5'-GAG AGA GAG ATC AAT GCC TTG TGT GAA-3',
	Reverse 5'-CGG ATT GGT GAC TAA AGT TGA TCC AAA-3'

CD49b	Forward 5'-CCC TGT GGA CCT ACC CAC TGC-3',
	Reverse 5'-TGA GGG TCA ATC CCA GGC TCA-3'

CD49c	Forward 5'-GGG AGC CTG TCG GAC CAC AT-3',
	Reverse 5'-AAA GCG CAG GGT CCA ACA CA-3'

CD49d	Forward 5'-TGG AGA AAA TTT TGC ATC ATG TCA AGC-3',
	Reverse 5'-CGG TGC CAG TCC AGT ACG ATG-3'

CD49e	Forward 5'-TTG GGG GAC CTG GAC CAA GA-3',
	Reverse 5'-TGG GCC TCC CGG GAA TAT AAA-3'

CD49f	Forward 5'-CGG GAG CCT CTT CGG CTT CT-3'
	Reverse 5'-CTG CAG CGG GAG TGC TTC TG-3'

CD41	Forward 5'-GTC GCG CCA ACA CCA TGA G-3'
	Reverse 5'-GGG TCA CCG CCA AGC TGA AG-3'

CD51	Forward 5'-CCA CCA GTG GTT TGG AGC CTC T-3'
	Reverse 5'-TCC AAC TGG CTC TCT CTC CTG CT-3'

CD61	Forward 5'-TGA CGC CAT CAT GCA GGC TA-3'
	Reverse 5'-TGG GTC TTG GCA TCC GTG GT-3'

bFGF	Forward 5'-TGT GTG CCA ACC GGT ACC TT-3'
	Reverse 5'-TTC CAG TCG TTC AAA GAA GAA ACA-3'

GM-CSF	Forward 5'-CAT GCC TGT CAC GTT GAA TGA AGA-3'
	Reverse 5'-TCA GGC GGG TCT GCA CAC AT-3'

GAPDH	Forward 5'-CCT GCA CCA CCA ACT GCT TA-3'
	Reverse 5'-CAT GAG TCC TTC CAC GAT ACC A-3'

Heparanase	Forward 5'-GGG GCC GGA TGG ATT ACT TT-3'
	Reverse 5' -CCA TGA AAA ACC CGT CTC CA-3'

HGF	Forward 5'-GGG ACG GTA TCC ATC ACT AAG A-3'
	Reverse 5'-CTT TAC CGC GAT AGC TCG AA-3'

MMP2	Forward 5'-GCA TCG CTC AGA TCC GTG GT-3'
	Reverse 5'-GAA TGT GGC CAC CAG CAA GG-3'

MMP9	Forward 5'-TAA AGG CCG CTC GGA TGG TT-3'
	Reverse 5'-CCA ACT ACG GTC GCG TCC AC-3'

PIGF	Forward 5'-CCT AGC TGG GTT GGC TGT GCA T-3'
	Reverse 5'-GCT GCG ACC CCA CAC TTC GT-3'

TWEAK	Forward 5'-CCG AGC TAT TGC AGC CCA TT-3'
	Reverse 5'-GCC ACT CAC TGT CCC ATC CA-3'

VEGFa164	Forward 5'-GCC AGC ACA TAG AGA GAA TGA GC-3'
	Reverse 5'-CAA GGC TCA CAG TGA TTT TCT GG-3'

VEGF-B	Forward 5'-CGC CTG CTG CTT GTT GCA CT-3'
	Reverse 5'-TCC ATG GCA CCA CTT TCT TCT GG-3'

VEGFR1	Forward 5'-TCA TGC AAG CAG GCC AGA CTC TC-3'
	Reverse 5'-CCT TTT GTC CTC CTG GCT CAC G-3'

VEGFR2	Forward 5'-GGG CTT GAT TTC ACC TGGC ACT C-3'
	Reverse 5'-CGC CAC AGT CCC AGG AAA GG-3'

### Immunohistochemistry

Solid tumor samples were snap-frozen in OCT medium (Tissue Tek, Sakura, Torrance, CA) and sections were prepared using a Leica CM1950 Cryostat (Leica Microsystems, Bannockburn, IL). Sections were fixed in cold acetone for 10 minutes, pretreated with 3% H2O2 for 20 min to block endogenous peroxidase activity and blocked in normal horse serum (Vector Laboratories). Biotinylated rat anti-mouse CD11c (HL3) and biotinylated hamster isotype control (both BD Pharmingen) were used at 1:50 dilution for these studies. Then, the Vectastain ABC kit was applied as described by the manufacturer (Vector Laboratories). Sections were counterstained with Gill's hematoxylin (Vector Laboratories). Images were acquired through a Micropublisher 5.0 Digital CCD Color Camera (Qimaging, Surrey, BC Canada).

### ELISA analysis

The concentration of different cytokines in culture supernatants was quantified by antigen capture ELISA. We used the following purified antibodies for capture: anti-mouse IL-1α (ALF-161), IL-6 (MP5-20F3) (both eBioscience, San Diego, CA) and anti-mouse-VEGF (BAF493, R&D Systems). For detection, we used biotin anti-mouse IL-1 α (Polyclonal), IL-6 (MP5-32C11) and biotin anti-mouse VEGF (AF-493-NA, R&D Systems) at 1 μg/well. Standard curves were constructed using recombinant murine IL-1α (220-11), IL-6 (216-16) and VEGF (450-32) (all Peprotech). Murine FGF was evaluated in culture supernatants by using the Human FGF-basic ELISA Development Kit (Peprotech) which cross-react 100% with mouse. Each dilution of recombinant standard or sample was assayed in duplicate. The reaction was developed by using streptavidin-horseradish peroxidase (554066, BD Pharmingen) and the 2,2'-azino-di-[3-ethylbenzthiazoline sulfonate(6)] (ABTS) substrate system (Roche Diagnostics GmbH, Mannheim, Germany). The blue-green color produced by enzymatic activity was quantified at 405 nm in an ELISA microplate reader (Multiskan RC, ThermoLabsystems).

### Proliferation assays

Murine myeloid C57BL/6 DCs recovered from long-term cultures and treated for 48 h with an inflammatory cocktail as described above were seeded in 96-well round-bottom plates at a concentration of 1 × 10^5^/well in RPMI containing 10% FBS. Spleens were resected from healthy BALB/c mice and minced in a sterile fashion to yield a single cell suspension and erythrocytes were eliminated by hypotonic shock. Then untouched CD3 T cells were purified from this suspension by magnetic sorting using the Pan T Cell Isolation Kit (MACS Miltenyi) following the manufacturer's instructions. T cells were labeled with CFSE as previously described [25] and were incubated at a concentration of 1 × 10^5 ^cells/well with the recovered DCs for 5 days. CFSE dilution, an indication of cell proliferation, was assessed by qualitative flow cytometry analysis of gated CD3 T cells.

### Statistical analysis

For multiple comparisons we performed ANOVA analysis with post-analysis by the Tukey-Kramer multiple comparisons test or the Dunnett's post comparison test. A value of p < 0.05 was considered significant. Data are expressed as mean ± SD. Data was analyzed by using the Graph Pad Instat software (GraphPad Software, Inc., San Diego, CA).

## Results

### Expression of adhesion molecules by myeloid DCs

**Figure 1:**
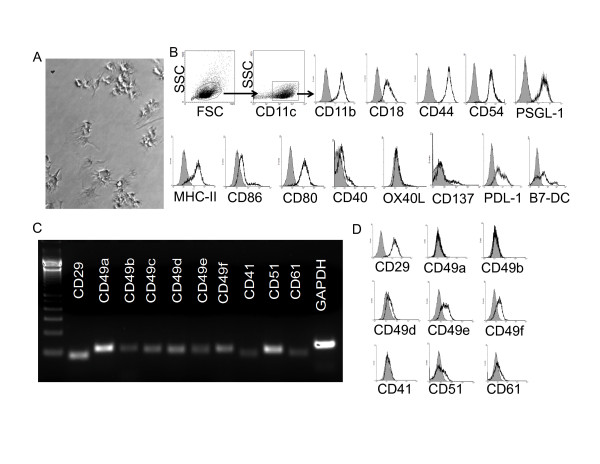
**Immature mDCs express an array of adhesion molecules capable of interacting with ECM components**. (**A**) Microphotograph of mDCs upon differentiation from bone marrow precursors (20 × magnification). (**B**) Qualitative flow cytometry analysis of costimulatory molecules and adhesion molecules to leukocytes or endothelial cells in immature myeloid DCs. Grey histograms represent isotype controls. An experiment representative of 3 independent experiments is depicted. (**C**) PCR qualitative analysis of adhesion molecules to ECM components in immature mDCs. An experiment representative of 3 independent experiments is shown. (**D**) Qualitative flow cytometry analysis of adhesion molecules to ECM components in immature myeloid DCs. Grey histograms represent isotype controls. An experiment representative of 3 independent experiments is depicted.

In the studies reported herewith, we used murine bone marrow-derived (myeloid) DCs. Figure 1A depicts myeloid DCs showing typical dendrites at the end of their 8-day differentiation culture from bone marrow precursors. As determined by qualitative flow cytometry analysis (Figure 1B), these cells are characterized by the expression of CD45 (not shown), β2 integrins CD11c and CD18, and the myeloid marker CD11b while lacking expression of CD8α molecule (not shown) usually present in murine splenic DCs. We also investigated the expression of MHC-II and members of the costimulatory B7/CD28 (B7-1/2[CD80, CD86], and PDL-1/2) and the TNF/TNF receptor (CD40, OX40L, and CD137) families. As shown in Figure 1B, the immature cells express low to null levels of surface costimulatory molecules CD86, CD40 and CD137 when compared to the isotype controls, while expressing higher levels of MHC-II, CD80, PDL-1 and PDL-2/B7-DC. All these molecules are upregulated upon activation (not shown). These molecules can participate either in activation (CD80, CD86, CD40, CD137, and OX40L) or suppression (PDL1/2) of T cell activity [26], being the final effect a result of the interplay between these sets of costimulators and coinhibitors. In addition, by means of flow cytometry analysis we were also able to detect, in the surface of these cells, the expression of different adhesion molecules that participate in interactions with other leukocytes or endothelial cells such as CD44, CD54 or PSGL-1 [27,28].

Murine myeloid(m) DCs have been extensively used in order to determine the efficacy and improvement of DC-based vaccines; investigate DC:T cell interactions or DC development; and determine their role in pathological conditions such cancer or infectious diseases [29-36]. Thus, we decided to use them as a model for our studies. We have previously reported that these cells exhibit high plasticity, being capable of acquiring angiogenic properties *in vivo *under pathological conditions [12]. We hypothesized that this might be caused not only by the presence of specific cytokines or growth factors in their microenvironment, but also by their physical interaction with different surfaces such as those generated by deposition of extracellular matrix (ECM) components. To investigate this we performed a series of studies in order to determine the influence of adhesion surfaces on the biology of DCs. First, by means of qualitative PCR analysis we studied at the level of RNA the expression of adhesion molecules capable of interacting with ECM components. In particular we investigated the expression of CD29 (binds with CD49 isoforms to generate adhesion complexes), CD49a (CD49a\CD29 complex binds to laminin and collagen), CD49b (CD49b\CD29 complex binds to collagen and laminin), CD49c (CD29\CD49c complex binds to laminin, collagen, fibronectin and thrombospondin), CD49d (CD49d\CD29 complex binds to fibronectin, and cellular components VCAM and MadCam), CD49e (CD49e\CD29 complex binds to fibronectin), CD49f (CD49f\CD29 complex binds to laminin), CD41 and CD61 (CD41/CD61 complex binds to fibrinogen, fibronectin and vitronectin), and CD51 (CD51/CD61 complex mediates adhesion to fibrinogen, fibronectin, vitronectin and thrombospondin) [37]. As shown in Figure 1C, we were able to detect expression of these molecules in mDCs at the level or RNA. In a series of complementary studies, we decided to investigate the expression of these molecules at the level of protein. To accomplish this we performed a direct staining of surface mDC molecules using fluorescent antibodies and we analyzed the cells qualitatively by means of flow cytometry. As shown in Figure 1D, immature mDCs express detectable levels of surface CD29, CD49d, CD49e, CD49f and CD51 at the level of protein when compared to the isotype controls. Contrary to what we observed at the level of RNA, we detected very low to null expression of CD49a, CD41 and CD61 in the same cells by flow cytometry analysis using direct staining. We cannot discard that these molecules are expressed at very low levels on the surface of the cells and require more sensitive methods, such as indirect staining, for their detection by flow cytometry.

Altogether these data indicate that similar to what has been reported in the human [38-40], murine mDCs express an array of adhesion molecules capable of interacting with ECM components.

### Generation of DC cultures on different surfaces and media

We have previously shown that murine mDCs could be cultured on Matrigel in the presence of tumor factors for long periods of time [12]. Similar studies were performed with human DCs grown on fibronectin and treated with tumor factors [41]. In order to investigate the role of different surfaces on the biology of mDCs, we cultured these cells on plates coated with fibronectin, collagen I, gelatin, Matrigel, poly-D-Lysine (a synthetic polymer which displays uniform net positive charges on the adhesion surface), or regular plastic culture surfaces (polystyrene) for up to 3 weeks. Also, to determine the effect of different environmental conditions, we cultured these cells in regular DC medium (RPMI) or endothelial cell growth medium (EGM). Regular DC medium consisted of RPMI medium supplemented with 10% FBS. The EGM endothelial medium is supplemented with 10% FBS together with several growth factors such as (human (h)EGF, hydrocortisone, hVEGF, hFGF-B, hIGF-1, ascorbic acid) that support endothelial cell proliferation.

**Figure 2:**
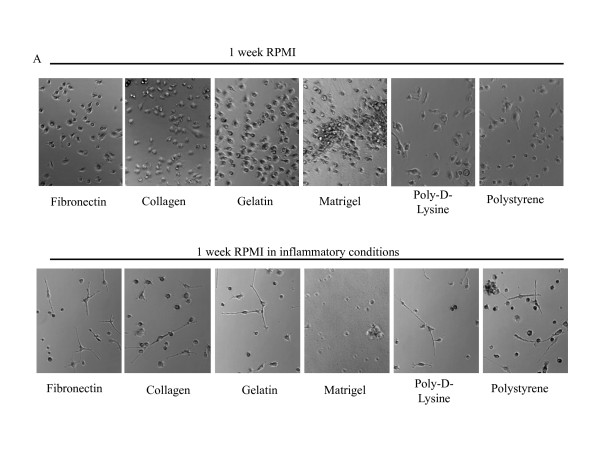
**Qualitative analysis of 1-week mDC cultures on different surfaces**. Representative microphotograph of mDCs after 1 week of culture with RPMI on different surfaces in the absence (**A**) or presence (**B**) of 100 ng of LPS and 20 ng/ml TNFα (20 × magnification). Two to 5 independent experiments were performed.

**Figure 3:**
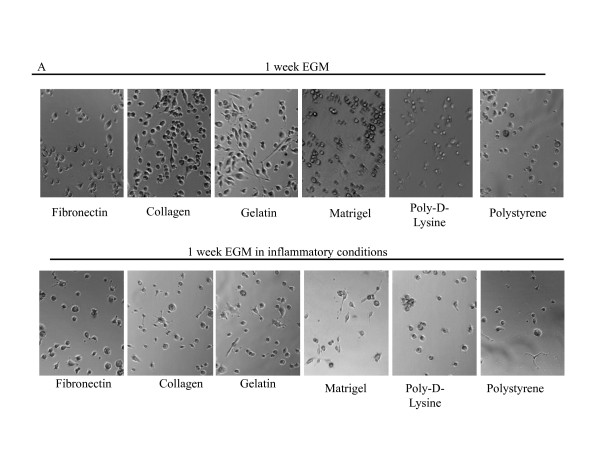
**Qualitative analysis of 1-week mDC cultures on different surfaces**. Representative microphotograph of mDCs after 1 week of culture with EGM on different surfaces in the absence (**A**) or presence (**B**) of 100 ng of LPS and 20 ng/ml TNFα (20 × magnification). Two to 5 independent experiments were performed.

Upon one week of culture in different conditions we performed a qualitative photographic analysis of the attached mDCs. As shown in Figure 2A, mDCs cultured for a week with RPMI on different surfaces in the absence of any maturation stimuli did not show the typical dendritic prolongations usually depicted by these cells (Figure 1A). On the contrary, when cultured in the presence of an inflammatory cocktail composed by 100 ng/ml of LPS + 20 ng/ml of TNFα, we observed a dramatic decrease in the number attached cells and also a change in the shape of the cells that remained attached, a great proportion of which showed an elongated shape (Figure 2B). Interestingly, this was not observed when cells were grown on Matrigel-coated surfaces. Similarly, when cultured 1 week with EGM, DCs also lose their typical shape (Figure 3A), but the effects of inflammatory stimuli on the morphology and attachment of these cells was less pronounced that when cells were cultured in the presence of RPMI (Figure 3B).

### Phenotypical analysis of DCs upon culture on different surfaces and media

**Figure 4:**
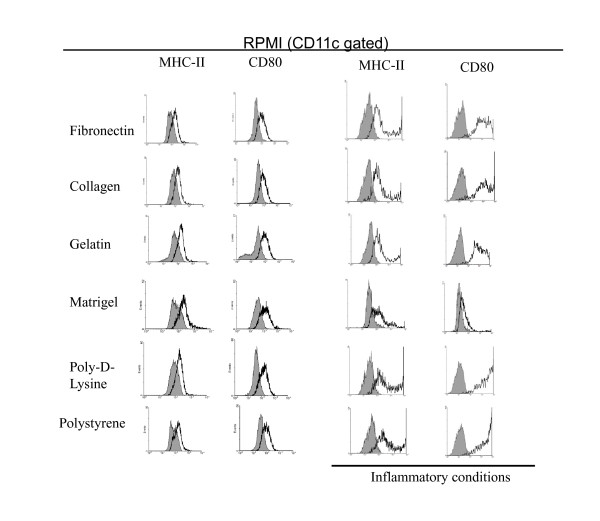
**Expression of surface markers on mDCs cultured for 1 week on different surfaces with RPMI media in the presence or absence of inflammatory factors**. Cells were recovered from different cultures and analyzed by qualitative flow cytometry. Analysis was performed on CD11c gated cells. Grey histograms represent isotype controls. The experiment was repeated 2 to 5 times with similar results.

**Figure 5:**
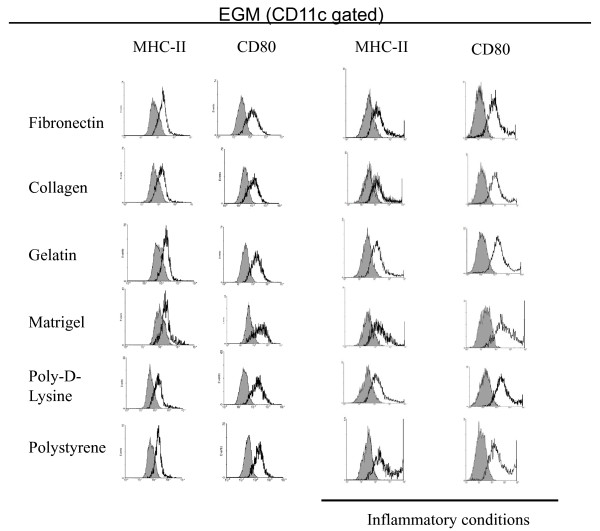
**Expression of surface markers on mDCs cultured for 1 week on different surfaces with EGM media in the presence or absence of inflammatory factors**. Cells were recovered from different cultures and analyzed by qualitative flow cytometry. Analysis was performed on CD11c gated cells. Grey histograms represent isotype controls. The experiment was repeated 2 to 5 times with similar results.

In order to further determine the effect of different culture conditions on the biology of mDCs, we evaluated the expression of costimulatory molecules by qualitative flow cytometry analysis on CD11c gated cells. As depicted in Figure 4, mDCs cultured with RPMI on different surfaces for 1 week in non-inflammatory conditions showed lower expression of costimulatory molecules CD80 and MHC-II as compared with the original population (Figure 1B). When the cells were cultured in the presence of an inflammatory cocktail they upregulated the expression of MHC-II and CD80 molecules. This was most evident when mDCs were cultured on poly-D-lysine and polystyrene substrates. This suggests that ECM components are able to impair the effect of maturation factors on the expression of costimulatory molecules by attached mDCs. Further, cells cultured on Matrigel, which showed little change in their morphology when treated with inflammatory molecules, showed no upregulation of costimulatory molecules as determined by flow cytometry analysis. Similarly, mDCs cultured with EGM also showed low levels of costimulatory molecules, but contrary to what happened when cells were cultured with RPMI, we were not able to observe upregulation of costimulatory molecules in the presence of inflammatory molecules (Figure 5).

**Figure 6:**
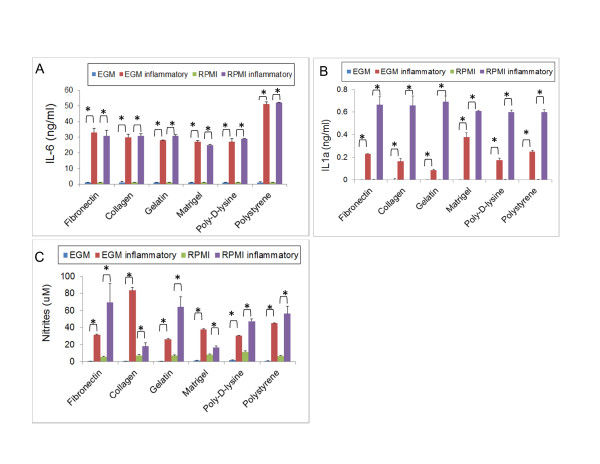
**One-week mDC cultures response to inflammatory stimuli**. IL-6 (**A**), IL-1α (**B**) and nitrites (**C**) were detected by ELISA analysis on mDC-cultures after 1 week culture in the presence or absence of 100 ng/ml LPS and 20 ng/ml TNFα. An experiment representative of two independent experiments with n = 2 for each condition is shown. (**A**) IL-6 production by DC cultures. All inflammatory stimuli-treated samples showed significantly higher levels of nitrites when compared to their untreated counterparts as determined by ANOVA analysis followed by Tukey-Kramer Multiple Comparisons post-test. *p < 0.001. (**B**) IL-1α production by DC cultures. All inflammatory stimuli-treated samples showed significantly higher levels of nitrites when compared to their untreated counterparts as determined by ANOVA analysis followed by Tukey-Kramer Multiple Comparisons post-test. *p < 0.001. (**C**) Nitrite detection by the Griess method. All inflammatory stimuli-treated samples showed significantly higher levels of nitrites when compared to their untreated counterparts as determined by ANOVA analysis followed by Tukey-Kramer Multiple Comparisons post-test. *p < 0.001.

In a series of complementary studies, we investigated the supernatants of these cells for inflammatory cytokines and nitrites. Upon treatment with LPS and TNFα, mDCs are able to generate inflammatory molecules such as IL-6 and IL-1α, and to upregulate the expression of nitric oxide synthase-II, which generates nitric oxide. As shown in Figure 6A-C, mDCs cultures treated with an inflammatory cocktail generate statistically significant higher levels of IL-6, IL-1α and nitrites (a subproduct of nitric oxide metabolism) than untreated controls. Altogether, these data indicate that these mDCs retained their capability to respond to immune stimulation by producing inflammatory molecules, even though they showed low levels of costimulatory molecules (i.e. in the presence of EGM).

### Production of angiogenic factors by 1-week mDC cultures

**Figure 7:**
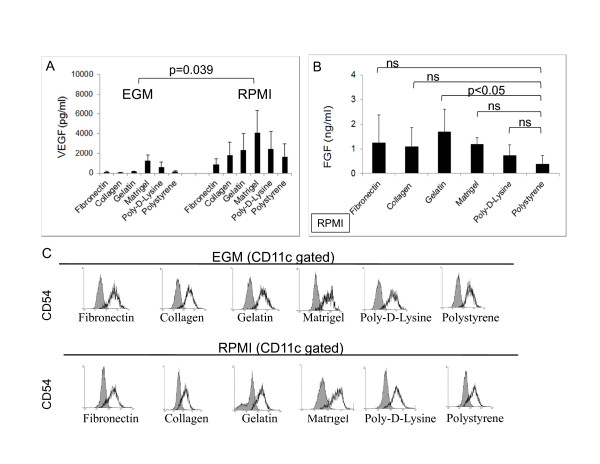
**One-week mDC cultures express angiogenic molecules**. (**A**) VEGF was detected by ELISA analysis on mDC-culture supernatants. The ELISA assay specifically recognized murine VEGF. Data from 5 independent experiments (n = 2 for each condition in every experiment) were pooled for this analysis. No significant differences were obtained in the EGM or RPMI groups as determined by ANOVA analysis. However the EGM group as a whole showed significantly lower VEGF levels than the RPMI group as determined by ANOVA analysis. (**B**) FGF ELISA analysis of RPMI cultures. Variable expression of this molecule was observed in repeated experiments. Being the ELISA assay able to recognize human FGF, a component of the EGM media, only RPMI supernatants were evaluated. Data from 5 independent experiments (n = 2 for each condition in every experiment) were pooled for this analysis. Only gelatin cultures showed significantly higher levels of FGF when compared to the polystyrene control as determined by ANOVA analysis followed by Dunnett's post comparison test. (**C**) CD54 expression in 1-week cultures. A qualitative flow cytometry analysis was performed in order to determine the expression of CD54 in the surface of these cells. The experiment was repeated 3 times with similar results. Grey histograms represent isotype controls.

Taking into account previous data showing that DCs associated with pathological conditions are able to produce angiogenic factors [13,41] we decided to investigate the presence in our cultures of VEGF and basic fibroblast growth factor (FGF) which have been reported to participate in angiogenic processes [42-45]. In multiple experiments we were always able to detect in all our cultures the presence of murine VEGF by means of ELISA analysis (Figure 7A). Although we were not able to detect significant differences in VEGF levels among the different surfaces conditions within the EGM or RPMI group (non-significant as determined by ANOVA analysis followed by Tukey-Kramer Multiple Comparisons) we were able to determine that as a whole, the RPMI cultures expressed higher VEGF levels than the EGM groups (p = 0.039). We considered that this is due to the presence of human VEGF in EGM cultures which can interact with murine VEGF receptors [46] and might downregulate the expression of this molecule by these cells. We also investigated the presence of FGF in our cultures. In this case, since the ELISA kit used for our studies is able to detect the human FGF present in the EGM medium, only samples taken from RPMI cultures were used. As shown in Figure 7B, we detected the expression of FGF in 1-week mDCs cultures. As determined by ANOVA analysis, only cells cultured in Matrigel generate significantly higher levels of FGF (p < 0.05) than the polystyrene control. Altogether these data indicate that mDC cultures are able to generate angiogenic factors.

Finally, DCs cultured for one week on different surfaces and media conditions express CD54 as determined by comparison with isotype controls in a qualitative flow cytometry assay (Figure 7C). This molecule has a crucial role in the clustering of DCs with lymphocytes by interacting with LFA-1 on the surface of T cells [47]. This indicates that these mDCs are able to interact with T cells although not being capable of positively activating them due to low levels of costimulatory molecules.

### Relevance of GM-CSF on the phenotype of DC cultures

**Figure 8:**
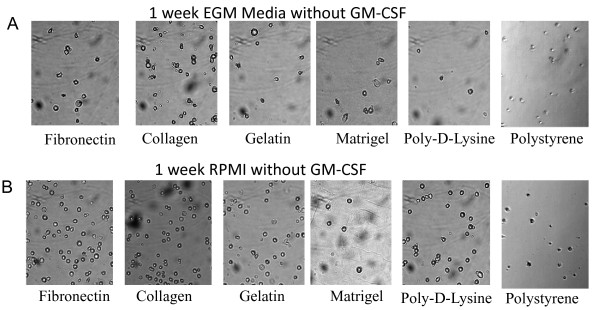
**Qualitative analysis of mDC attachment to different surfaces in the absence of GM-CSF**. Microphotograph of mDCs after 1 week of culture on different surfaces in EGM (**A**) and (**B**) RPMI in the absence of GM-CSF. The experiment was repeated 4 times with similar results. (20 × magnification)

**Figure 9:**
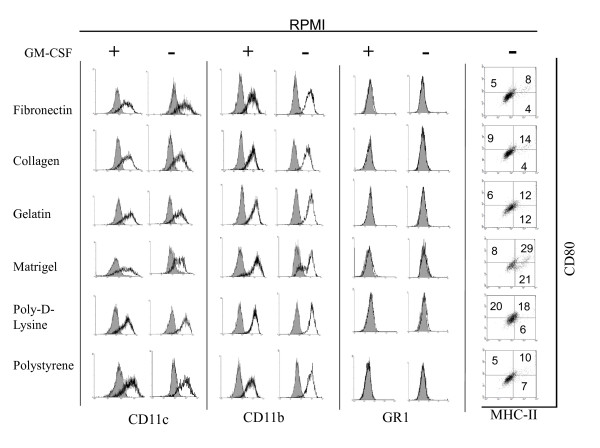
**GM-CSF and mDC expression of surface molecules by cells cultured with RPMI**. Myeloid DCs were cultured for 1 week on different surfaces in RPMI in the presence or absence of 3 ng/ml of GM-CSF. Attached cells were recovered and the expression of CD11c, CD11b, GR1, MHC-II and CD80 analyzed by qualitative flow cytometry. An experiment representative of 2 to 5 independent experiments is shown. Grey histograms represent isotype controls. Quadrants in dot plot graphs were defined using isotype controls.

**Figure 10:**
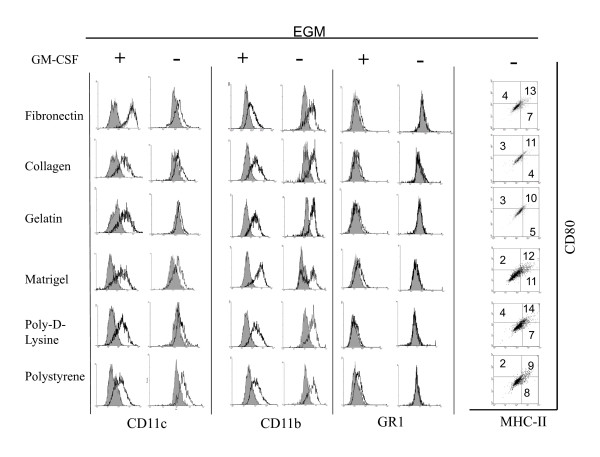
**GM-CSF and mDC expression of surface molecules by cells cultured with EGM**. Myeloid DCs were cultured for 1 week on different surfaces in EGM in the presence or absence of 3 ng/ml of GM-CSF. Attached cells were recovered and the expression of CD11c, CD11b, GR1, MHC-II and CD80 analyzed by qualitative flow cytometry. An experiment representative of 2 to 5 independent experiments is shown. Grey histograms represent isotype controls. Quadrants in dot plot graphs were defined using isotype controls.

In our previous experiments both RPMI and EGM cultures were supplemented with external GM-CSF. Myeloid DCs obtained *in vitro *through the method described by Lutz [16] are differentiated from bone marrow precursors in the presence of GM-CSF (20 ng/ml). In our studies, after 1 week of culture in the absence of GM-CSF few mDCs remained attached to the different surfaces (Figure 8A and 8B) as determined by qualitative micro-photographic analysis. These cells did not spread on the culture surface as their GM-CSF-treated counterparts, but rather adopt a circular shape. Further, it was not possible to generate 2 or 3-weeks cultures with enough attached cells for analysis. Then, we decided to investigate the expression of different surface immune molecules in these cells by qualitative flow cytometry analysis. In particular, we analyzed the expression of CD11c, CD11b and GR1, and costimulatory molecules MHC-II and CD80 in 1-week RPMI and EGM cultures supplemented or not with GM-CSF. As described by Lutz [16], when mDC cultures are extended for more than 10 days after bone marrow collection, a decrease in the levels of GM-CSF is recommended. For our studies we used a GM-CSF concentration of 3 ng/ml [16]. As depicted in Figure 9, mDCs cultured with RPMI in the presence or absence of GM-CSF showed little modification in the expression of CD11c and CD11b as determined by qualitative flow cytometry analysis. On the other hand, the expression of costimulatory molecules was low in the absence of GM-CSF as determined by dot plot analysis of MHC-II and CD80 markers. The proportion of cells expressing simultaneously both markers ranged from 8 to 29% in different conditions, as determined by quadrants defined using isotype controls. Similar results were obtained when cells were cultured with EGM in the absence of GM-CSF. It is interesting to comment that mDCs cultured with EGM in the presence or absence of GM-CSF decreased the levels of CD11b expression, a typical myeloid marker (Figure 10) when compared with similar cells cultured with RPMI (Figure 9). Further, cells cultured in the presence of EGM also showed a decrease in the expression of CD11c (Figure 10). This was more evident when GM-CSF was not present in the culture medium. This suggests that in a context such as the tumor microenvironment which is rich in angiogenic factors, cells that were originally mDCs, might lose particular markers, thus being overlooked when the tumor-associated leukocyte population is analyzed. Furthermore, only in cells cultured with EGM we were able to detect the expression of GR1 beyond control isotype levels, thus indicating that part of the original mDC population could now co-express CD11b and GR1 markers, typical of myeloid derived suppressor cells, which have been detected in tumor settings orchestrating antitumor immune responses [48]. On the other hand, no GR1 expression was observed when mDCs were cultured with RPMI, independently of the surface assayed (Figure 9) as determined by qualitative flow cytometry analysis.

### Generation of GM-CSF by ovarian cancer cells and its effect on mDC phenotype

**Figure 11:**
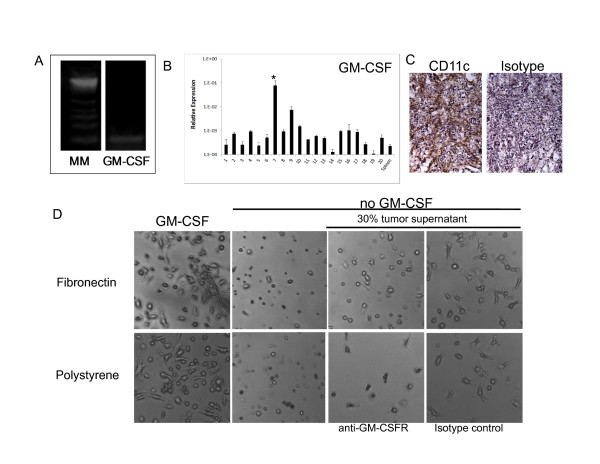
**Tumor-derived GM-CSF and mDC cultures**. (**A**) Expression of GM-CSF by mouse ovarian tumor cells at the level of RNA as determined by qualitative PCR. An experiment representative of 2 independent experiments is shown. (**B**) Expression of GM-CSF by several mouse ovarian tumors at the level of RNA as determined by quantitative real time PCR. Samples were analyzed in quadruplicate. All samples except tumor 7 (* p < 0.05) expressed GM-CSF at levels that were not significantly different from those detected in spleen, an immune organ that was used as a control for these studies. (**C**) Staining of mouse ovarian tumors shows heavy DC infiltration. Immunohistochemistry analysis (200 × magnification). A photograph representative of 8 stained tumors is depicted. (**D**) Myeloid DCs were cultured on polystyrene and fibronectin in the presence of RPMI supplemented or not with GM-CSF, and RPMI supplemented with 30% tumor conditioned medium without addition of GM-CSF in the presence of anti-GM-CSF receptor antibody (4 μg/ml) or isotype control. (20 × magnification). An experiment representative of 2 independent experiments with n = 2 is shown.

**Figure 12:**
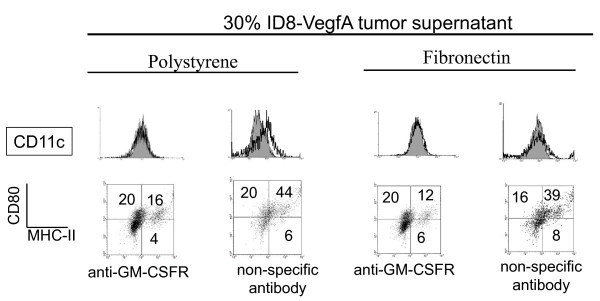
**Expression of surface markers on mDCs cultured for 1 week in the presence of tumor factors**. Myeloid DCs were cultured on polystyrene and fibronectin in the presence of RPMI medium supplemented with 30% tumor conditioned medium without addition of external GM-CSF and in the presence of anti-GM-CSF receptor antibody (4 μg/ml) or isotype control. Cells were recovered from cultures after 1 week and analyzed by qualitative flow cytometry. Grey histograms represent isotype controls. Quadrants in dot plot graphs were defined using isotype controls. An experiment representative of 2 independent experiments (n = 2 each experiment) is shown.

We have previously reported that mDCs can be cultured for more than one week in ovarian cancer conditioned medium [12]. We speculated that the tumor medium could be a source of GM-CSF which can promote survival of mDCs. Thus, we examined the capability of mouse ovarian cancer cells to produce GM-CSF. As shown in Figure 11A, mouse ovarian cancer cells expressed GM-CSF at the level of RNA as determined by qualitative PCR analysis. Further, we decided to investigate if whole tumors could be a source of GM-CSF *in vivo*. To accomplish this, mouse ovarian tumors were developed in C57BL/6 mice, dissected and RNA was extracted from whole tumor samples and analyzed by real-time quantitative PCR. As determined by ANOVA analysis followed by Dunnett post-comparison tests, we were able to determine that all tumor samples except one expressed GM-CSF at levels that were not significantly different from those observed in normal murine spleen, an immunological organ used as a control in these studies (Figure 11B). Production of GM-CSF by mouse ovarian tumors might help interpret the heavy DC infiltration observed in this tumor model (Figure 11C) [12]. To further investigate the relevance of tumor-derived GM-CSF on mDCs we cultured these cells with supernatants containing 30% of tumor conditioned medium (30% v/v; ID8-VegfA tumor conditioned medium:RPMI). Since tumor conditioned medium harbors other growth factors such as M-CSF and G-CSF (not shown), as a control we treated some cultures with anti-GM-CSF receptor antibody as previously described [22]. mDCs cultured with RPMI plus 3 ng/ml of GM-CSF on fibronectin and polystyrene attached and spread on these surfaces (Figure 11D) as determined by qualitative micro-photographic analysis. As described above, this phenotype is lost in the absence of GM-CSF. Cells cultured with tumor conditioned medium in the absence of externally added GM-CSF attached and spread on both surfaces, showing a phenotype that is close to those of cells cultured with normal medium in the presence of GM-CSF. When an anti-GM-CSF receptor antibody was added on day 1 of culture, we were able to detect a lower number of attached cells, which showed a phenotype that resembled those cells cultured in normal medium in the absence of GM-CSF as determined by qualitative micro-photographic analysis. These data indicate that GM-CSF present in the tumor condition medium contributes to promote attaching and spreading of mDCs on different surfaces. Similar to what happened when these cells were cultured in EGM, tumor conditioned medium abrogated the expression of CD11c by mDCs to isotype levels as determined by qualitative flow cytometry analysis (Figure 12).

Similarly, blocking the effect of tumor-derived GM-CSF decreased the levels of costimulatory molecules in these cells, as determined when comparing the proportion of cells expressing both markers in anti-GM-CSFR treated and control samples. This indicates that the presence of this molecule in the tumor microenvironment might help preserve mDC function.

### Angiogenic properties of long-term DC cultures

**Figure 13:**
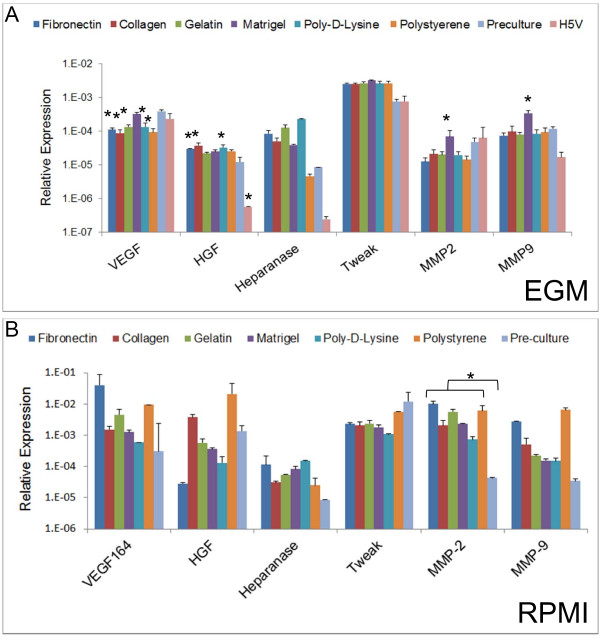
**Expression of angiogenic molecules from 3-week mDC cultures**. DCs were recovered from different cultures after 3 weeks in EGM (**A**) or RPMI (**B**), RNA was extracted and reverse-transcribed. Then, quantitative real-time PCR was performed to analyze several angiogenic molecules in these cells. (**A**) As determined by ANOVA analysis followed by Dunnett multiple comparison test using pre-culture mDCs as a control, expression of VEGF, HGF, MMP2 and MMP9 was significantly different under some conditions (* p < 0.05). (**B**) MMP-2 expression levels in the RPMI cultured-group showed statistically significant differences respect to pre-culture mDCs at p < 0.05 as determined by ANOVA analysis. For **A **and **B**, samples were run in duplicate in each experiment and further analyzed in duplicate by quantitative real-time PCR. An experiment representative of 2 independent experiments is shown.

In this series of studies we decided to investigate if after 3 week in culture mDCs were able to produce angiogenic factors. To accomplish this we analyzed, by means of real-time PCR, the expression of several angiogenic molecules previously described in APCs. In particular, we evaluated the expression of VEGF-A 164 (which is the predominant VEGF-A isoform) [44], hepatocyte growth factor (HGF) [49], Heparanase [50], TWEAK [51,52] and matrix metalloproteases (MMPs) 2 and 9 [53]. As controls we used the same cells before culture (pre-culture DCs) and we also include for comparison H5V, a murine endothelial cell line [54]. As shown in Figure 13A, cells cultured in the presence of EGM on fibronectin, collagen, gelatin, poly-D-lysine and polystyrene showed a significant decrease in the expression of VEGF as compared with pre-culture DCs (ANOVA analysis followed by Dunnett post comparison test). This was not observed when cells were cultured with RPMI (Figure 13B). This correlates with the decrease in VEGF protein levels observed in one week EGM cultures when compared to RPMI cultures (Figure 7A). We were also able to detect an increase in HGF RNA levels in mDCs cultured with EGM on fibronectin, collagen and poly-D-lysine. Finally, EGM cultures in Matrigel also expressed significantly higher levels of MMP2 and MMP9 at the level of RNA. On the other hand, upon ANOVA analysis we were not able to detect almost any significant differences respect to pre-culture mDCs on cells cultured with RPMI. The only exception being the expression of MMP-2, whose expression was significantly higher (p < 0.05) in every condition when compared to pre-culture cells. Finally, we decided to include H5V, a murine endothelial, cell line for comparison in our studies. As shown in Figure 13A, the expression of several angiogenic molecules by H5V was similar to what was detected in pre-culture DCs, being the exception the expression of HGF which was significantly lower in these endothelial cells. This similar behavior between H5V and pre-culture mDCs highlights the angiogenic potential of these APCs.

**Figure 14:**
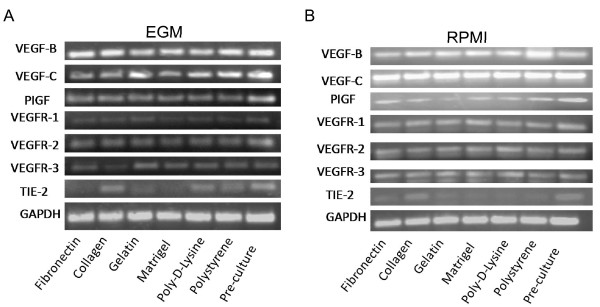
**Qualitative PCR analysis of angiogenic molecules expression by 3-week mDC cultures**. DCs were recovered from different cultures after 3 weeks in EGM (**A**) or RPMI (**B**), RNA extracted and reverse-transcribed. Then, qualitative PCR was performed to analyze angiogenic molecules and receptors in these cells. An experiment representative of 2 independent experiments is shown.

In a complementary series of studies, we analyzed by qualitative PCR the presence of additional angiogenic factors and receptors in these cells. As shown in Figure 14A and 14B, we detected expression of VEGF B and C, and PIGF in all our samples, even on pre-cultured DCs at the level of RNA. Further, these cells expressed VEGFR-1 and -2 at the level of RNA (receptors for VEGF 164, VEGF-B and PIGF) suggesting that they can be involved in autocrine loops. Finally, TIE-2, a receptor for angiopoietin and associated with angiogenic leukocytes [55] was detected at the level of RNA in pre-cultured cells and in some of the cultured cells.

### Immunological capabilities of DCs upon long-term cultures

**Figure 15:**
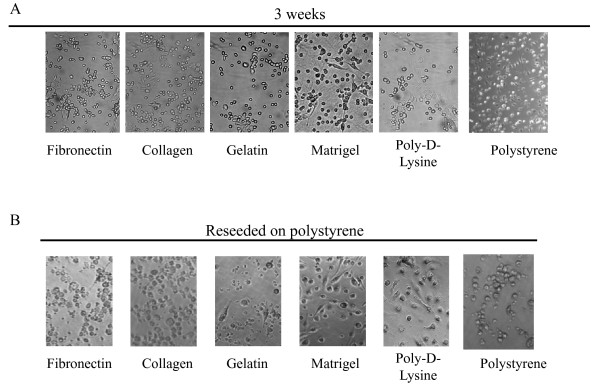
**Qualitative analysis of mDCs upon reattachment to plastic**. (**A**) Microphotograph of mDCs after 3 weeks of culture on different surfaces (20 × magnification). (**B**) Microphotograph of reattached DCs. Myeloid DCs cultured for 3 weeks on different surfaces with EGM were detached, purified using CD11c magnetic beads and cultured on polystyrene for 24 h (20 × magnification). An experiment representative of 2 independent experiments is shown.

**Figure 16:**
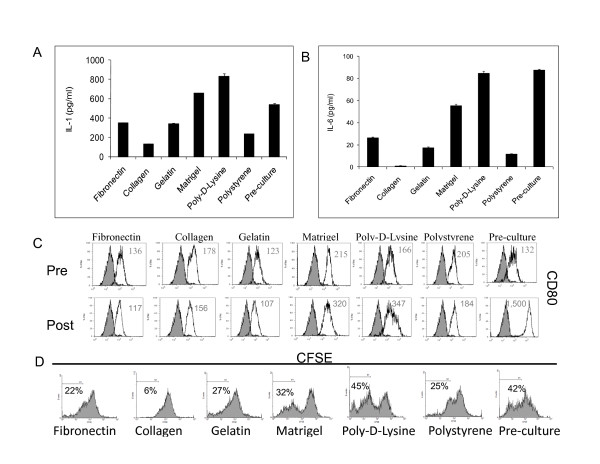
**Immunological properties of 3-week mDC cultures in EGM**. IL-1α (**A**) and IL-6 (**B**) were detected by ELISA on reseeded cultures after 48 h stimulation with TNFα and LPS. (**A**) ANOVA analysis: p < 0.0001. Tukey-Kramer Multiple Comparisons post-Test: Fibronectin vs. collagen, Matrigel, poly-d-lysine, polystyrene and preculture: p < 0.001; vs. gelatin: NS. Collagen vs. gelatin, Matrigel, poly-d-lysine, polystyrene, and preculture: p < 0.001. Gelatin vs. Matrigel, poly-d-lysine, polystyrene, and preculture: p < 0.001. Matrigel vs. poly-d-lysine, polystyrene, and preculture: p < 0.001. Poly-d-lysine vs. polystyrene, and preculture: p < 0.001. Polystyrene vs. preculture: p < 0.001. An experiment representative of 2 independent experiments is shown. (**B**) ANOVA analysis: p < 0.0001. Tukey-Kramer Multiple Comparisons post-test: Fibronectin vs. collagen, Matrigel, poly-d-lysine, polystyrene, and preculture: p < 0.001; vs. gelatin p < 0.01. Collagen vs. gelatin, Matrigel, poly-d-lysine, polystyrene, and preculture: p < 0.001. Gelatin vs. Matrigel, poly-d-lysine, and preculture: p < 0.001; vs. polystyrene: p < 0.05. Matrigel vs. polystyrene, poly-d-lysine, and preculture: p < 0.001. Poly-d-lysine vs. polystyrene: p < 0.001; vs. preculture: NS. Polystyrene vs. preculture: p < 0.001. An experiment representative of 2 independent experiments is shown. An experiment representative of 2 independent experiments is shown. (**C**) Qualitative flow cytometry analysis of CD80 in mDCs recovered from 3-week cultures before and after treatment with inflammatory factors. Grey histograms represent isotype controls. Numbers represent mean fluorescence intensity values. An experiment representative of 2 independent experiments is shown. (**D**). CFSE dilution analysis. Proliferation of CFSE-stained allogeneic BALB/c lymphocytes was determined by qualitative flow cytometry analysis after 5 day co-culture with the same mDCs as in (**C**). An experiment representative of 2 independent experiments is shown. Numbers represent percent of CFSE negative cells.

Herewith we decided to focus our studies on the immune properties of mDCs cultured for 3 weeks with EGM on different surfaces. The rationale being that in previous studies endothelial medium seemed to skew the cells farther away from a typical immunostimulatory DC phenotype [56,57]. As shown in Figure 15A, in some of the experimental conditions, 3-week DC cultures on different surfaces with EGM medium showed little morphological resemblance with the original population (Figure 1A) as determined by qualitative micro-photographic analysis. In order to define the immunological properties of these mDCs we isolated CD11c positive cells from the cultures by means of magnetic sorting and cultured equal amounts of live cells in regular culture plastic plates (polystyrene) for 24 h (Figure 15B) before inducing maturation. To accomplish this, cultures were treated with a typical inflammatory cocktail (100 ng/ml of LPS + 20 ng/ml of TNFα) for 48 h. As shown in Figure 16A and 16B, we were able to detect different levels of inflammatory cytokines IL-1α and IL-6 in our cultures in response to stimulation. In particular, very low levels of those molecules were produced by CD11c cells recovered from collagen cultures, while CD11c recovered from Matrigel or poly-D-Lysine cultures produced cytokine levels similar to those obtained by fresh DCs. In the same way, when analyzing the expression of CD80, a typical costimulatory molecule, we observed that only CD11c cells recovered from Matrigel and poly-D-Lysine cultures seemed to upregulate the expression of this molecule (Figure 16C) as determined by qualitative flow cytometry analysis.

To further determine the immune capabilities of these cells, after stimulation with an inflammatory cocktail we cocultured them with CFSE-stained allogeneic BALB/c T lymphocytes. Consistently with the ELISA and FACS data, CD11c cells recovered from Matrigel and poly-D-Lysine cultures induce higher levels of proliferation of allogeneic T cells than cells recovered from other conditions (Figure 16D). Indeed, CD11c recovered from collagen cultures, which produce the lowest levels of inflammatory cytokines upon stimulation, were unable to induce proliferation of T cells as determined by qualitative flow cytometry analysis. Altogether, these data indicate that the interaction with different surfaces affect in different ways the immunological properties of mDCs.

## Discussion

DCs present in peripheral tissues sample the organism for the presence of antigens, which they take up, process and present in their surface in the context of MHC molecules. Typically, antigen-loaded DCs migrate to immunological organs where they present the processed antigens to T lymphocytes thus triggering specific immune responses. On the other hand, a role of DCs as promoters of angiogenesis under pathological conditions has been suggested [13,15,58,59]. Our hypothesis is that in the tumor microenvironment, this phenotypic shift is caused by the combined effect of cytokine/growth factor signaling and interaction with ECM components.

We and others have shown that monocytes or DCs can undergo an endothelization process *in vitro *characterized by the loss of CD14/CD45 and upregulation of endothelial markers such as CD31, CD34, von Willebrand factor, VEGF receptor (VEGFR)-2 and VE-Cadherin [12,41,60]. These cells can display other characteristics of *bonafide *endothelium such as LDL uptake, lectin binding or formation of cord-like structures in 3D gels [12,24,41,58,60,61]. DCs can also promote angiogenesis by generating angiogenic factors, i.e., DCs with proangiogenic properties have been described in tumors and other pathological conditions [15,58,62-64]. These data indicate that DCs have a high plasticity, reacting to their microenvironment by changing their morphology and biological activity. This becomes important taking into account that it has been postulated that the microenvironment where they encounter their antigenic stimuli will define the outcome of the DC:T cell interaction (Signal 3) and the consequent immune response.

Several reports indicate that soluble factors can alter the biology of DCs. Tumor-associated cytokines such as VEGF, IL-10 and PGE-2 can profoundly affect the nature of APCs. In particular, DCs showing low levels of costimulatory molecules have been detected in tumors expressing high levels of VEGF [65] and tumor patients treated with anti-VEGF antibody showed a decrease in the levels of immunosuppressive DCs [66]. In the same way, it has been shown that the endothelial cell-produced antiangiogenic cytokine vascular endothelial growth inhibitor induces DC maturation [10]. Another molecule present in the tumor microenvironment that can profoundly affect DC biology is PGE-2. This molecule has been shown to induce the production of IL-10 and VEGF by human DCs [8].

On the other hand, few studies have been undertaken in order to determine the effect of different ECM components on the biology of DCs. Foundational work on this subject was performed on 1998 by Brand *et al. *[39]. This research group, working with human monocyte-derived DCs, investigated the adhesion of these cells to collagen I, IV and fibronectin. They were able to show that these cells adhere differentially to these ECM components, rapidly changing their morphology when they attached to fibronectin or increasing their maturation status upon interaction with collagen I. In another series of experiments, Ammon *et al. *[38] described the expression of different integrin receptors in human monocytes, monocyte-derived DCs and macrophages. More recently Kohl *et al.*, [40] reported that human DCs derived from CD34 precursors or from monocytes differentially bind to ECM components, and display different expression of surface integrins.

Herewith we report that attachment to a surface together with factors provided in the media allowed long-term mDC cultures to exhibit different immunological capabilities. DCs are usually considered short-lived, terminal cells whose main function is to activate T cells in order to induce specific immune responses. We were able to demonstrate that these cells need the presence of GM-CSF in order to be able to establish long-term cultures; that GM-CSF can be produced by ovarian cancer cells and that is expressed in the microenvironment of mouse ovarian tumors. In terms of the role of adhesion surfaces on the biology of these cells, we were able to show that cells cultured on different surfaces showed expression of angiogenic molecules such as HGF, bFGF, MMP2 and MMP9. We also showed that these cells are able to produce VEGF-A, -B and PIGF. Taking into account that most of these molecules were expressed in every condition it is tempting to speculate that if mDCs are not driven to initiate an immune response they will by default remain in a proangiogenic state. Further, since we were able to detect the expression of VEGFR-1 and -2 in these cells it is tempting to speculate that some of these angiogenic molecules might act in an autocrine fashion, promoting cell survival or induction of other angiogenic molecules. Finally, expression of MMPs by DCs might promote VEGF availability [67] thus contributing to angiogenic process but might also help degrade ECM components facilitating metastasis in a tumor setting.

In terms of immunological properties, we observed that after 1 week culture in non-inflammatory conditions, mDCs showed low levels of costimulatory molecules. Attachment to the different surfaces did not inhibit the capability of these cells to respond to inflammatory stimuli. In particular, these cells upregulated the expression of costimulatory molecules, and produced IL-1α, IL-6 and nitric oxide when cultured in the presence of LPS and TNFα. This effect was less pronounced when cells were cultured with EGM, indicating that the factors present in this medium can decrease the effects of inflammatory factors. Further, DCs cultured on different surfaces for 3 weeks showed differential immunological capabilities. In particular, DCs recovered from Matrigel, (a gelatinous protein mixture purified from murine tumor stroma) and from poly-D-Lysine (an artificial polymer) were able to respond to maturation stimuli similarly to fresh mDCs. Upon stimulation, these cells produced large quantities of IL-1α and IL-6, and induced proliferation of allogeneic lymphocytes. On the other end, DCs recovered from collagen I surfaces were completely unresponsive to maturation stimuli.

## Conclusions

Herewith we demonstrated that the combination of soluble factors together with adhesion surfaces determines particular mDC profiles. Thus, in order to design DC-based vaccines or treatments focused on changing the phenotype of DCs associated with diseases, such as cancer [68-70] or atherosclerosis [71,72] among others, it becomes necessary to fully investigate the microenvironment in which these cells are present or will be delivered.

## Authors' contributions

LS: ELISA analysis, qualitative and quantitative PCR; MM: qualitative and quantitative PCR: analysis, immunohistochemistry analysis; MP: ELISA analysis, qualitative and quantitative PCR, direction of coworkers; EM: qualitative PCR analysis; J.M: qualitative PCR analysis; HN: Immunohistochemistry, quantitative PCR; AKV: ELISA analysis; AR: ELISA Analysis MG and OO: Nitrite analysis; KM: qualitative PCR analysis; MCC: FACS analysis; FB: Cell culture, sample collection, *in vivo *studies, FACS analysis, direction of coworkers, experimental design and writing. All authors read and approved the manuscript.
